# An analytic, moment-based method to estimate orthopositronium lifetimes in positron annihilation lifetime spectroscopy measurements

**DOI:** 10.5604/01.3001.0054.9141

**Published:** 2024-12-22

**Authors:** Lucas Berens, Isaac Hsu, Chin-Tu Chen, Howard Halpern, Chien-Min Kao

**Affiliations:** 1 Department of Radiology, University of Chicago, Chicago, IL, USA; 2 Department of Radiation and Cellular Oncology, University of Chicago, Chicago, IL, USA; 3 Department of Computer Science, University of Minnesota, Minneapolis, MN, USA

**Keywords:** positronium, positron emission tomography, positron annihilation lifetime, moments, analytic

## Abstract

**Objective:**

The presence of tumor hypoxia is known to correlate with poor patient prognosis. Measurement of tissue oxygen concentration can be challenging, but recent advancements using positron annihilation lifetime spectroscopy (PALS) in three-dimensional positron emission tomography (PET) scans have shown promise for hypoxia detection. In this work, a novel method for estimating the orthopositronium lifetime in PALS is presented.

**Methods:**

We have developed an analytical method based on moments of the PALS histogram to estimate the orthopositronium lifetime. The method was characterized with respect to estimation bias and variance, and the bias-minimizing moments were found for various orthopositronium lifetimes.

**Results:**

For sufficient statistical power, the method produces monotonic, stable estimates. For cases with a lower number of photon counts, the method was characterized, and solutions are presented to correct for bias and estimation variability. For most cases, the bias-minimizing moments were n=10,11,12.

**Conclusions:**

This novel method does not require curve-fitting and thus it is less computationally intensive than methods which fit the PALS histogram. This method will be extended to and continue to be improved for use in positronium lifetime imaging.

## BRIEF DESCRIPTION OF THE WORK

We present an analytical method to estimate orthopositronium lifetimes in positron annihilation spectroscopy. This method can be extended to positronium lifetime imaging, for measurements of in vivo oxygen concentrations with PET scans.

## INTRODUCTION

Positronium lifetime imaging (PLI) is a novel augmentation of positron emission tomography (PET) that may allow PET scans to extract tissue oxygenation information, including hypoxic locations, in addition to the specific biochemical properties the employed PET tracer is responsive to. Recently, Moskal et al. has identified the positronium lifetime as a possible metric for hypoxia [1]. The same group has also designed and built a novel PET scanner for generating positronium lifetime images [2], a version of which has produced images from in vivo human brain data [3]. Other recent work includes that of Shibuya et al. in which the correlation of oxygen concentration with positronium lifetimes was rigorously established [4].

PLI makes use of measurements of the lifetime of positronium (Ps), which are short-lived bound states between a positron emitted from nuclear decay and an electron from the environment. In clinical PET circumstances, approximately 40% of the released positrons lead to this electron-positron bound state [4]. The other 60% annihilate with electrons without forming positronium. These produce direct annihilations (DA), and predominantly produce two coincident 511 keV photons with a lifetime of about 388 ps [2].

There are two types of positronium: orthopositronium (o-Ps), where the electron and positron spins are parallel, and parapositronium (p-Ps), where the spins are anti-parallel. Both are formed within 5 ps of the release of the positron after it has thermalized with the environment. In tissue, approximately 75% of positronium is o-Ps and 25% is p-Ps. Both o-Ps and p-Ps are inherently unstable and eventually their constituent electron and positron annihilate each other without interacting with their environment. In such self-annihilations, p-Ps produces two 511 keV photons^1^ and has a short lifetime of approximately 125 ps. On the other hand, o-Ps produces three coincidence photons and has a long lifetime of approximately 142 ns [5, 6].

As a result, o-Ps has the opportunity to interact with electrons that are present in its surroundings and to decay before self-annihilation. In biological systems the o-Ps lifetime is observed to be on the order of several nanoseconds [1]. These interactions include a spin-exchange with an unpaired electron that converts o-Ps to p-Ps where it quickly decays to produce two 511 keV photons, and a pick-off event where the positron annihilates with a local electron (also most likely to produce two 511 keV photons). In tissue, the average o-Ps lifetime is between 1–3 ns and importantly its value depends on local properties that affect the strength of the spin-exchange interaction [4]. For example, molecular oxygen is paramagnetic and can cause o-Ps decay, since o-Ps readily decays by interacting with unpaired electrons.

If we therefore choose an isotope that possesses a nontrivial decay mode in which a prompt gamma and a positron are released at essentially the same time, we may measure the arrival time difference between the prompt gamma and coincident 511 keV photons. A metric for local tissue oxygenation may then be derived. Specifically, the histogram S(Δt) of these differences in arrival times Δt can be modeled as the sum of multiple convolutions of exponential distributions and Gaussian distributions [7, 8]. We consider here the lifetime components from o-Ps, p-Ps, and DA as they have sufficiently distinct lifetimes and intensities to be modeled separately. Each is represented by one exponential distribution that models the probability distribution for the decay, convolved with a Gaussian distribution that models the statistical uncertainty in time measurement. Therefore, the full model for S(Δt) can be written as

(1) $$S(\Delta t)=b+\sum_{i=1}^{3}I_{i}\;\text{EXP}\left(\Delta t;\lambda_{i}\right)*𝒩\left(\Delta t-t_{0};\sigma_{i}\right)$$

where * denotes convolution, $I_{i},\lambda_{i}$, and $\sigma_{i}$ are the intensity, decay--rate constant, and standard deviation of the time-measurement uncertainty associated with component i for = 1,2,3, b≥0 accounts for the presence of background events, EXP is defined as

(2) $$\text{EXP}\left(t;\lambda \right)=\lambda \;\text{exp}\{-\lambda t\}\;u(t),$$

in which u(t) is the unit step function defined by u(t)=1 for t≥0 and u(t)=0 for t<0, and

(3) $$𝒩(t;\sigma )=(\sqrt{2\pi }\sigma {)}^{-1}\text{exp}\left\{-t^{2}/2\sigma^{2}\right\}.$$

The lifetime $\tau_{i}$ of component i is related to $\lambda_{i}$ by $\tau_{i}=1/\lambda_{i}$. In Equation 1, $t_{0}$ is introduced to allow for an offset in time measurement. We wish to estimate τ associated with o-Ps from a measurement of S(Δt) in providing a metric for certain tissue property. Typically, it is reasonable to take $\sigma_{i}=\sigma \forall i$ and $t_{0}=0$. Without loss of generality, we can take the components i=1,2,3 to be those from the p-Ps decay, DA, and o-Ps decay, respectively, and hence $\lambda_{1}>\lambda_{2}>\lambda_{3}$.

While PLI is emerging for the purpose of producing full three--dimensional images of the o-Ps lifetime, this work is concerned with only estimating the lifetime from a source by means of Equation 1. For distinction, we therefore refer to this method as one for positron annihilation lifetime spectroscopy (PALS) measurements. In the discussion we will comment on using this method for imaging in general and will consider it in-depth in a separate paper.

Presented here is a novel analytic method for estimating the o-Ps lifetime from S(Δt). Current methods for this task include fitting a single exponential distribution to the time difference histogram [4], and fitting a reduced full model given by Equation 1 by assuming known values for certain parameters to the histogram [2, 7, 8]. Additionally, Shibuya et al. has proposed an inverse Laplace transform method to distinguish between positronium lifetimes while merging voxels for better statistics [9]. Their method is able to discern similar lifetime values, but still employs curve-fitting. In comparison with these curve--fitting methods, the new analytic method in this work is more computationally efficient, which is an important consideration for future application of the method for PLI in which lifetimes need to be obtained for a large number of voxels in a clinical setting. In addition, under mild and realistic conditions the analytic method is not sensitive to the unknown lifetimes for DA and p-Ps, nor to the time-measurement uncertainty σ. Generally, this is the not case with curve-fitting methods.

## MATERIALS AND METHODS

### Derivation of the method and justifications

The nth moment of a function f(t) is defined by

(4) $$\mu_{n}\{f(t)\}=\int_{-\infty }^{\infty }t^{n}f(t)dt$$

if the integral exists. The proposed analytic method is based on the following observations.

1. The nth moment of EXP (t;λ) exists for all λ>0 and is given by
(5) $$\mu_{n}\{\text{EXP}(t;\lambda )\}=n!/\lambda^{n}.$$
2. Let g(t;λ,σ)=EXP(T;λ)*𝒩(t;σ). For sufficiently large λ (with respect to σ) and n, it can be shown that, for any s>0,
(6) $$\mu_{n}\left\{e^{-st}g(t;\lambda ,\sigma )\right\}\approx c(\lambda )\mu_{n}\{\text{EXP}(t;s+\lambda )\}$$
  where $\text{c}(\lambda )=[\lambda /(\text{s}+\lambda )]e^{{ơ}^{2}\lambda 2/2}$ is independent of n.
3. Applying the above to Equation 1 with $\sigma_{\text{i}}=\sigma$ and $t_{0}=0$ we get
(7) $$\mu_{n}\left\{e^{-s\Delta t}\tilde{S}(\Delta t)\right\}\approx n!\sum_{i=1}^{3}c\left(\lambda_{i}\right)I_{i}/{\left(s+\lambda_{i}\right)}^{n}$$
  where S(Δt)=(S(Δt)-b)⋅u(Δt).
4. Since $\lambda_{3}$ is assumed to be smaller than $\lambda_{1}$ and $\lambda_{2}$, the i=3 term dominates the sum in Equation 7 for large values of n, yielding
(8) $$R(n+1,s)\equiv \mu_{n+1}\left\{e^{-st}\tilde{S}(\Delta t)\right\}/\mu_{n}\left\{e^{-st}\tilde{S}(\Delta t)\right\}\approx (n+1)/\left(s+\lambda_{3}\right).$$

Therefore, with an adequately large n we have

(9) $$\tau_{3}\approx R(n+1,s)/[n+1-sR(n+1,s)+\delta ],$$

where the small positive value δ has been added to the denominator to control observed estimation variability for small values of $\tau_{3}$.

**Fig. 1.**
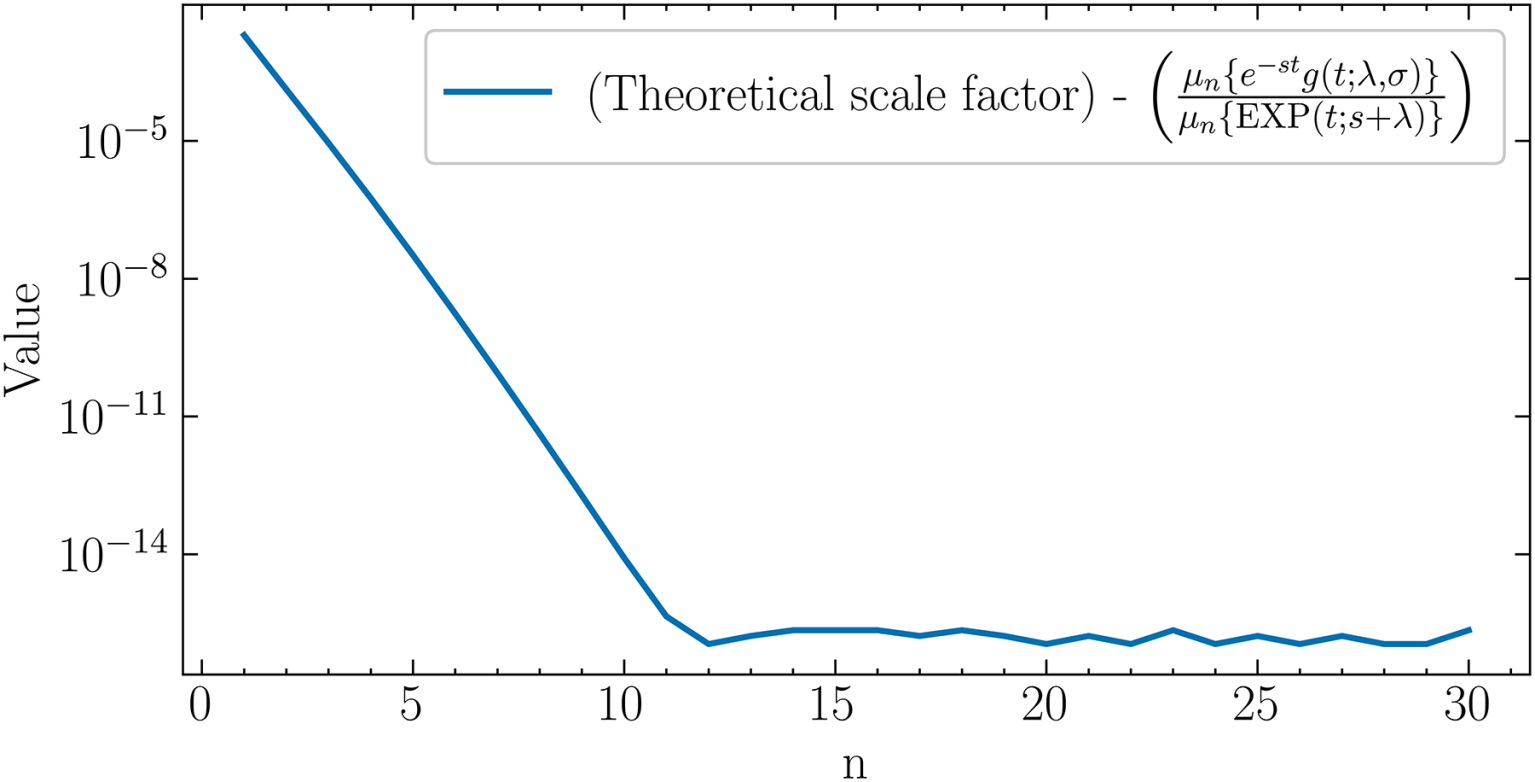
The scale factor c(λ) in Equation 6, from which the ratio $\mu \text{n}\left\{{\text{e}}^{-\text{st}}g(t;\lambda ,\sigma )\right\}\approx \text{c}(\lambda )/\mu_{n}\{\text{EXP}(t;s+\lambda )\}$ has been subtracted. Their difference has a maximum of 2.01 × 10^−3^ which decreases as n increases until n=12 whe the mean difference becomes 1.6 × 10^−16^. λ was set to 0.5 ns^−1^.

The plot in Fig. 1. demonstrates Equation 6 numerically for s=1 and several selected values for n. Derivations of Equation 5 and 6 are given in the Appendix.

For the constant values we used in Equation 1, we reference Moskal et al. [2]. These values are $\tau_{1}=1/\lambda_{1}\approx 0.125\;\text{ns}$ and $I_{1}\approx 0.1$ for p-Ps, $\tau_{2}=1/\lambda_{2}\approx 0.388\;\text{ns}$ and $I_{2}\approx 0.6$ for DA, and $\tau_{3}=1/\lambda_{3}\approx 1-10\;\text{ns}$ and $I_{3}\approx 0.3$ for o-Ps. The coincidence resolving time (CRT) was chosen to be 600 ps full width at half maximum (FWHM) from current state-of-the-art time-of-flight (TOF) PET systems [2, 10], yielding σ≤220ps^2^. Therefore, for PALS and PLI measurements the assumptions leading to Equation 9 can be verified.

In theory, $\tilde{S}(\Delta t)$ decreases exponentially to zero as Δt increases, which allows $\mu_{n}\{\tilde{S}(\Delta t)\}$ to be well defined. In reality, noise in $\tilde{S}(\Delta t)$ does not necessarily decrease with Δt and hence will contribute a substantial statistical error in $\mu_{n}\{\tilde{S}(\Delta t)\}$, especially for large n. This instability can be considerably reduced by instead using, $\mu_{n}\left\{{\text{e}}^{-s\Delta t}\tilde{\;\text{S}}(\Delta t)\right\},s>0$ as the term ${\text{e}}^{-s\Delta t}$ attenuates the contribution from data at large Δt. Although a large s is favored for alleviating the effects of noise, it also diminishes the differences among $\text{s}+\lambda_{ɨ}$ and thereby requires a large n for Equation 8 to hold true. However, calculation of higher-order moments is more susceptible to noise, so the value of s needs to be chosen with care. In this work, in consideration of the numerical values of $\lambda_{\text{i}}$ and $I_{\text{i}}$, we choose s=1 which is empirically justified based on the results to be reported below. Future work will consider s more extensively.

**Fig. 2.**
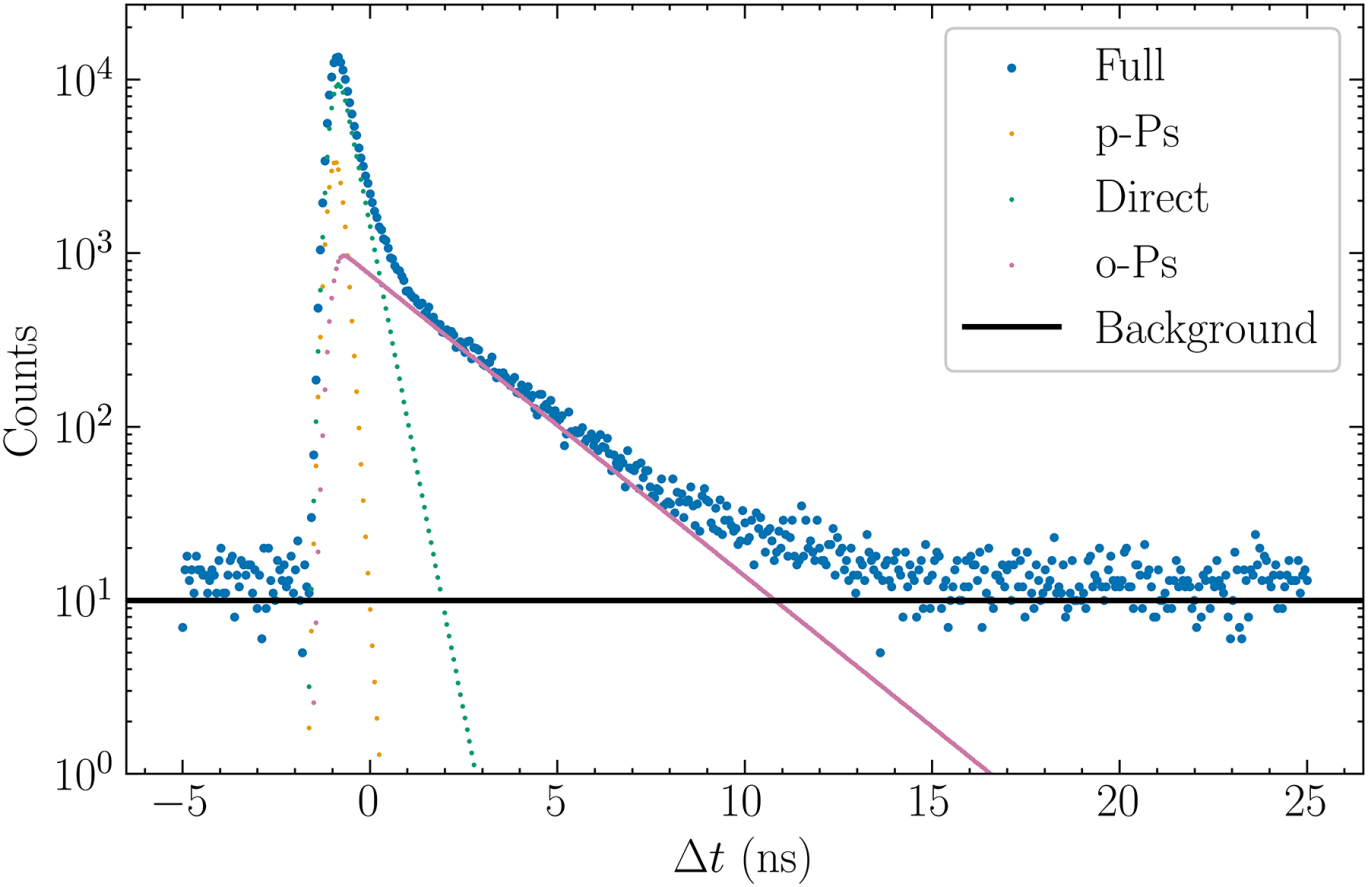
A simulated histogram of Δt with 2 × 10^5^ events and using a 60 ps bin size. It was generated by using $\tau_{1}I_{1}=0.078,\tau_{2}I_{2}=0.388,\tau_{3}I_{3}=0.165,\left(\lambda_{1}=8{\;\text{ns}}^{-1},\lambda_{2}=2.58{\;\text{ns}}^{-1}\right.,\left.\lambda_{3}=0.4{\;\text{ns}}^{-1}\right),b=10$, and $t_{0}=9\;\text{ns}$. The individual contributions from the p-Ps, DA, and o-Ps components are shown in yellow, green, and magenta, respectively.

From Equation 4, $\mu_{n}\{f(t)\}$ can be regarded as a filter that removes an increasingly wider range of small t data as n increases. By inspection of Fig. 2. it can be stated that by choosing an n which makes Equation 8 valid, a “soft” low cutoff has been introduced. This avoids using data where DA and p-Ps are dominant.

### Simulated data

The proposed method is evaluated by using computer generated simulation data. All computation codes were implemented in Python version 3.11, using the specified values for $b,I_{\text{i}},\lambda_{\text{i}}$, and σ, and the desired total number of events for the histogram N. The simulation program first computed $S\left(\Delta t_{j}\right)$ according to Equation 1 at discrete times in [−5 ns, 25 ns] and at a regular spacing of 60 ps. Next, it scaled $S\left(\Delta t_{j}\right)$ to yield $\sum_{\text{j}}S\left(\Delta t_{j}\right)=N$. Then the scaled $S\left(\Delta t_{j}\right)$ was replaced by an integer drawn by a Poisson random number generator (from the numpy.random module) whose mean equals the scaled $S\left(\Delta t_{j}\right)$. Fig. 2. shows an example of the generated histogram where the parameters were chosen so that it was similar to the measured histogram reported by Moskal et al. [2]. Each simulated histogram, except where noted, contained $N=2\times 10^{5}$ total events.

### Implementation and numerical studies

The background term b was estimated using the average of the histogram in the Δt<0ns region. The estimate was then subtracted from each histogram data points and $\lambda_{3}$ was calculated according to Equation 8 and Equation 9. For computing the moments, Equation 4 was approximated by a naive discrete summation: $\mu_{n}\left\{f\left(t\right)\right\}\approx \delta t\sum_{\text{j}}t_{\text{j}}^{\text{n}}\tilde{S}\left(t_{j}\right)$, where $t_{j}$ are the time points where f(t) is available and δt is the spacing of $t_{j}$.

The proposed method was evaluated for accuracy and precision against a number of parameters, including 1) the order of moment n used, 2) the upper truncation of the histogram data, 3) the number of counts N, and 4) the background level b. At present, the range of *in vivo* o-Ps lifetime values has not been precisely established. However, in a recent paper Moskal et al. observed that cardiac myxoma and adipose tissue had mean lifetimes of 1.912 ns and 2.613 ns, respectively [2]. Therefore, we performed evaluations for o-Ps lifetimes of 1.0, 1.5, 2.0, 2.5, and 3.0 ns to cover the likely *in vivo* lifetime range. On the other hand, since they are insensitive to the local environment [6], the reported mean values of 388 ps and 142 ps are used for DA and p-Ps lifetimes, respectively. For $I_{1},I_{2}$ and $I_{3}$, the values used were based on quantitatively matching the simulated and measured data.

## RESULTS

**Fig. 3.**
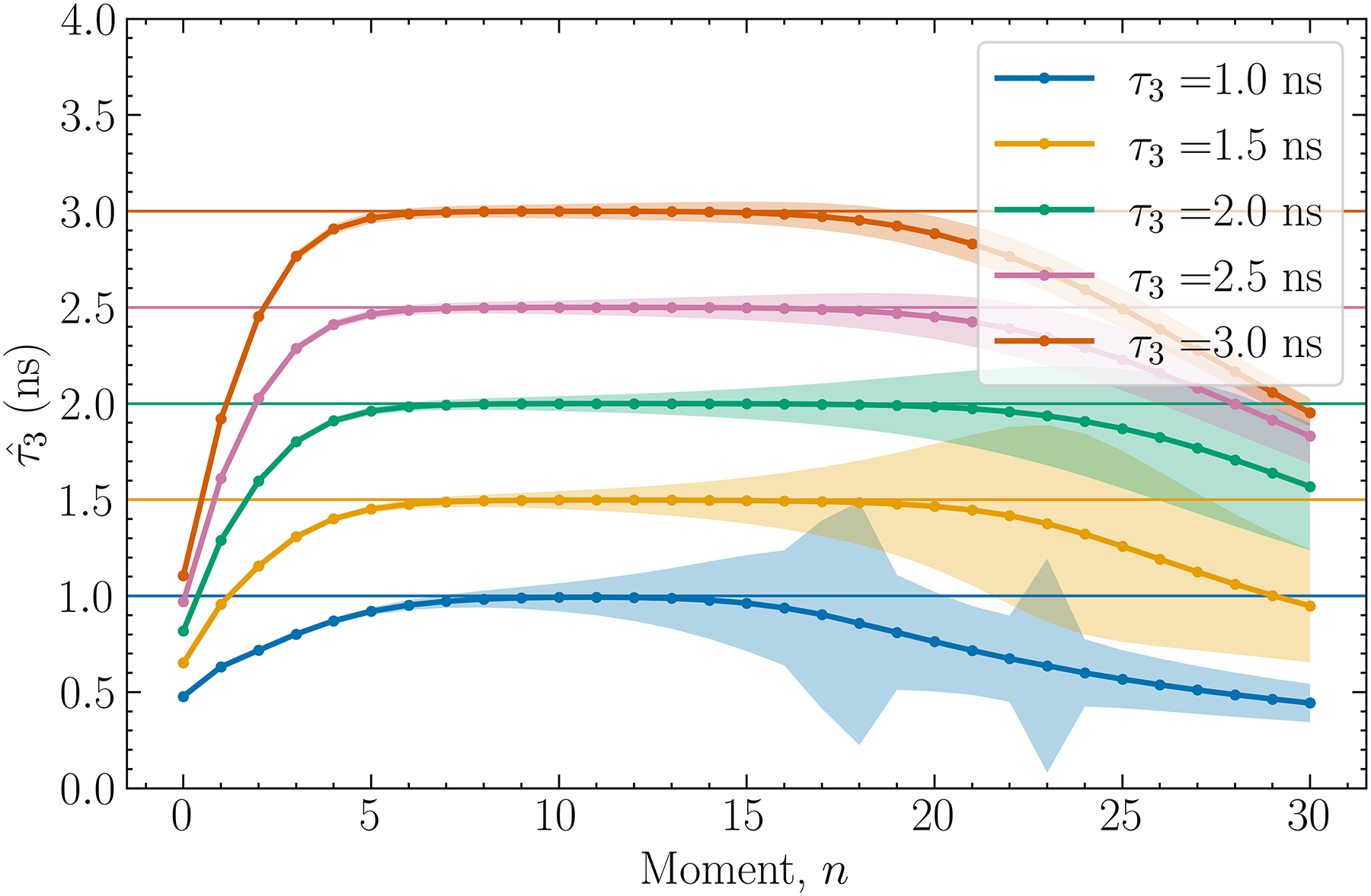
Estimated o-Ps lifetime as a function of the order of moment n. The shaded areas for each curve represent the ±1 standard deviation on each data point. The true $\tau_{3}$ values shown in the legend are those used for producing simulation data.

The results are presented by Fig. 3. through Fig. 10. Each figure contains individual points which are estimates of $\tau_{3}$, denoted by ${\overset{ˆ}{\tau }}_{3}$ below, derived from our method. Each data point is the mean of the results obtained from 1 × 10^4^ histograms simulated by using the same parameters (1 × 10^5^ in the case of Fig. 3.), and the shaded regions in the plots give the ±1 standard deviations (std) about the means. The horizontal lines, when present, indicate the true o-Ps lifetimes that are used to produce simulation data.

### Lifetime estimate versus order of moment n

Fig. 3. shows the estimated o-Ps lifetime when the order of moment n employed by Equation 9 is varied. Four general trends can be observed. First, all curves show a plateau where the estimated lifetime has essentially zero bias. This plateau occurs between n ≈ 5 and n ≈ 16 depending on the o-Ps lifetime. The standard deviations of the estimates are also sufficiently small to allow for discrimination of all the lifetimes examined. Second, the standard deviation increases with n, which is consistent with the observation made above that higher-order moments are more sensitive to data noise. Third, as $\tau_{3}$ increases the plateau occurs at a lower n.

This is because the differences between $s+\lambda_{3}$ and $s+\lambda_{\text{i}},i=1,2$ increases, allowing the i=3 term to dominate the sum in Equation 7 at a smaller n. Fourth, all curves decrease toward zero as the order increases. This is because higher-order moments are increasingly more contributed by data at larger Δt while data is simulated only for -5ns≤Δt≤25ns. In practice, the measured histogram is necessarily truncated.

It is also noted that at small n the estimated lifetime values are considerably smaller than the true values. This reflects the situation that with a small n the i=3 term no longer dominates the i=1,2 terms in Equation 7. As a result, Equation 9 yields approximately the same value since $I_{1,2}$ and $\tau_{1,2}$ are constants. Tab. I. summarizes the bias-minimizing order of moment in the plateau region and the statistics of the estimates obtained when using this order. As shown, the largest std/mean ratio is only 0.654%, obtained for $\tau_{3}=1.0\;\text{ns}$.

**Fig. 4.**
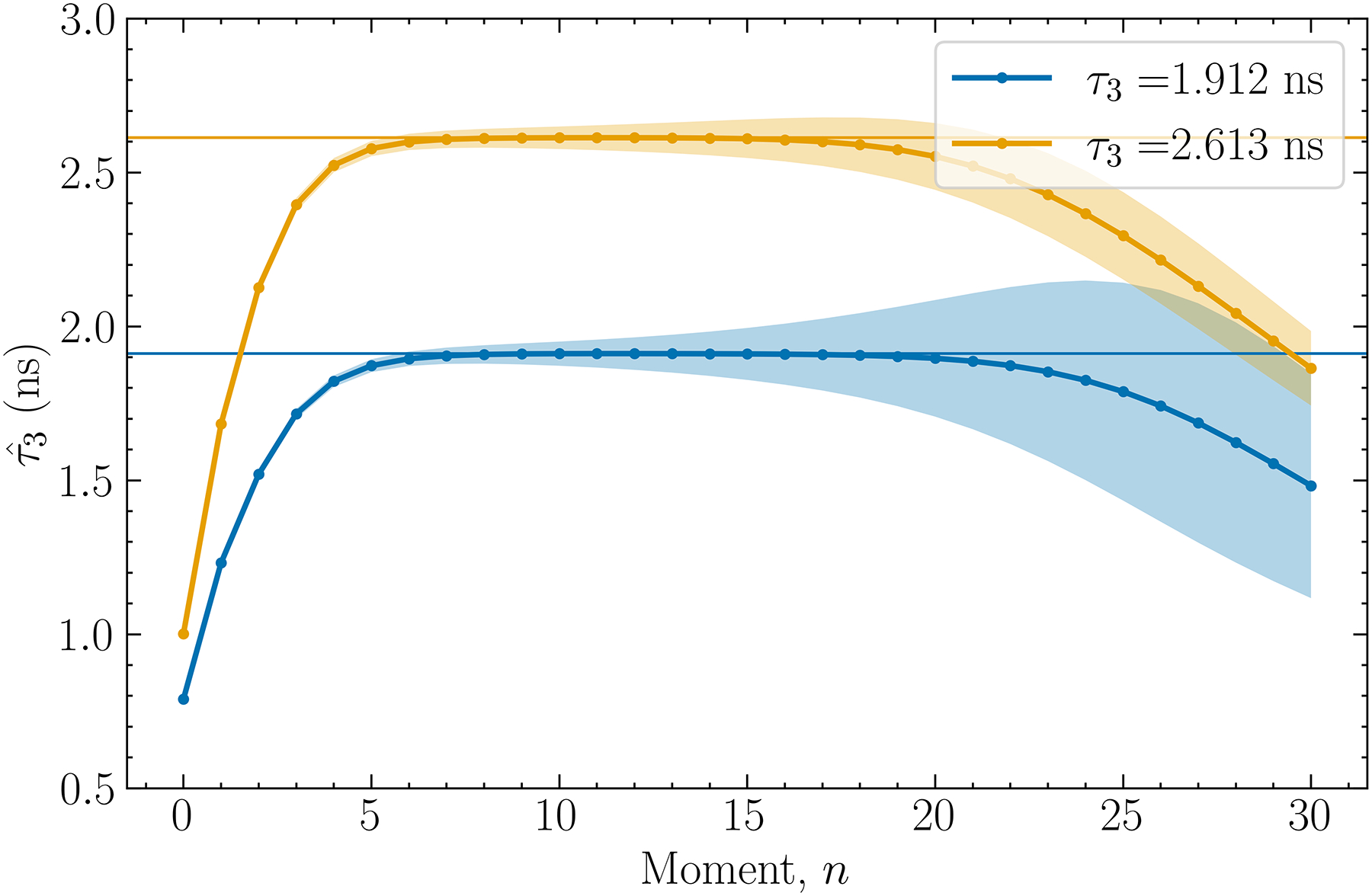
Estimated o-Ps lifetime as a function of the order of moment n, when modeling the time-difference histogram after *ex vivo* data from Moskal et al. [2]. The shaded areas for each curve represent the ±1 standard deviation on each data point.

Fig. 4. shows the result obtained when the mean $\tau_{3}$ values measured for cardiac myxoma and adipose tissue by Moskal et al. [2] were used to produce simulation data. In this case, the bias-minimizing orders of the moments were found to be n=11 for $\tau_{3}=1.912\;\text{ns}$ (myxoma tissue), which yielded ${\overset{ˆ}{\tau }}_{3}=1.91\pm 0.05\;\text{ns}$, and n=11 for $\tau_{3}=2.613\;\text{ns}$ (adipose tissue), which yielded ${\overset{ˆ}{\tau }}_{3}=2.61\pm 0.04\;\text{ns}$. The means of these estimates are within 0.02% and 0.005% of their respective true values.

### Lifetime estimate versus number of events N

**Fig.5.**
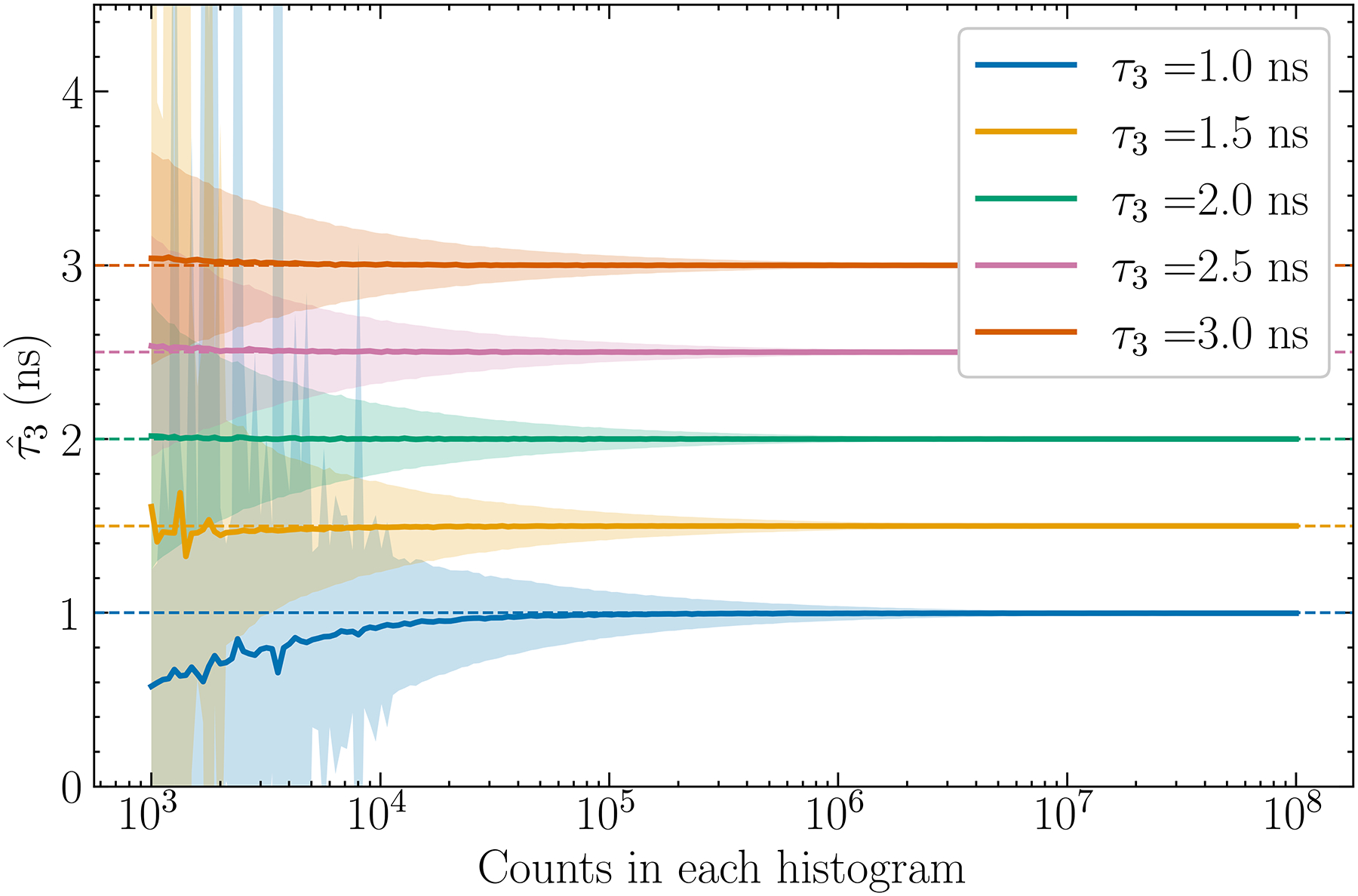
Estimated o-Ps lifetimes as a function of the number of events N in the histogram. The shaded areas again indicate ±1 standard deviation about the mean.

**Fig. 6.**
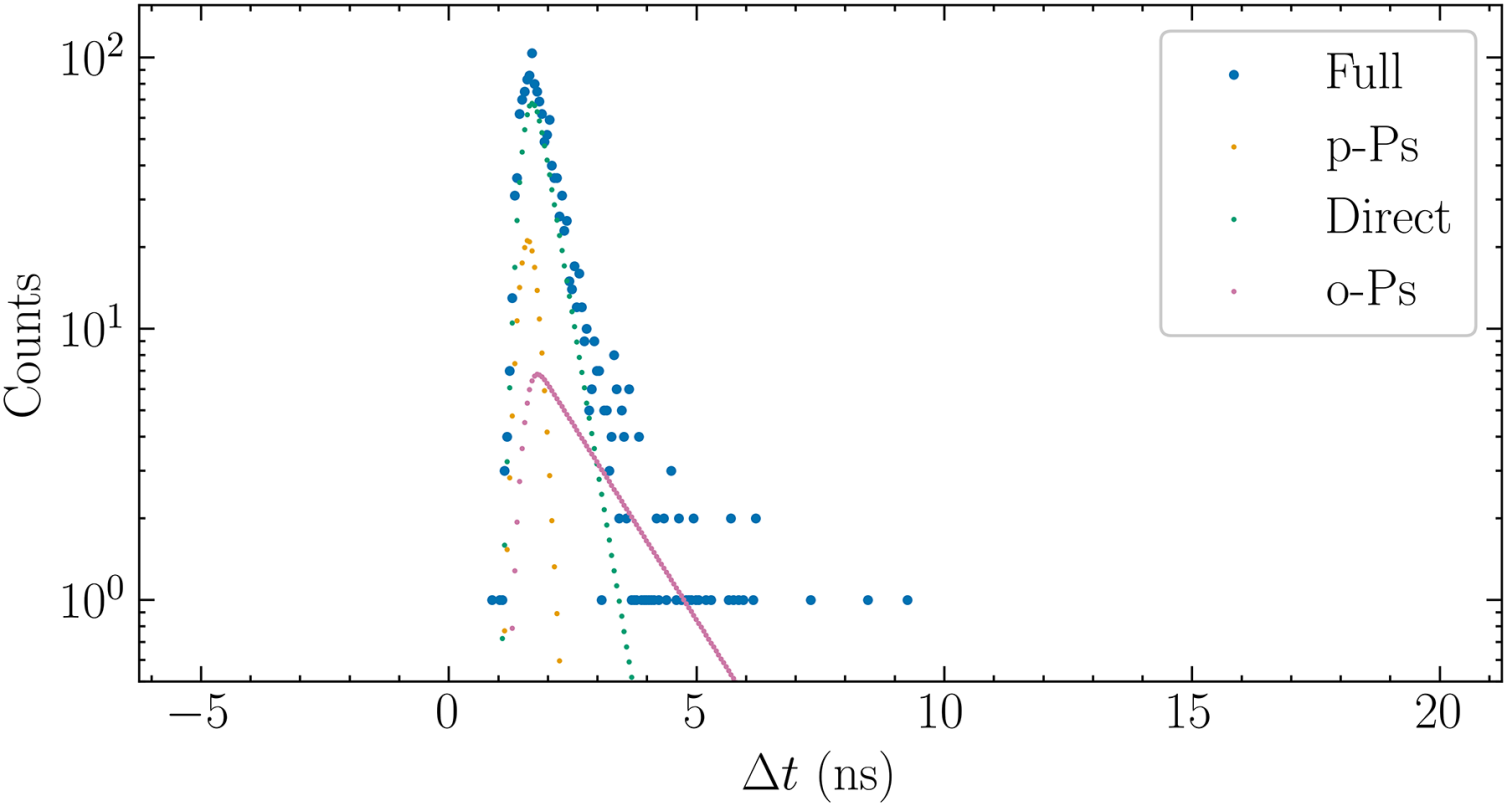
A simulated histogram for $\tau_{3}=1.5\;\text{ns}$ and N = 1,500, including the noise-free contributions of DA, p-Ps, and o-Ps.

The results discussed were obtained by using simulated data with $N=2\times 10^{5}$. Generally, a histogram derived from a larger number of events has better statistics and can lead to better estimates for $\lambda_{3}$. Fig. 5. shows the results for N ranging from 1×10^3^ and 1 × 10^8^. The order of moment was fixed to n=11. A tabulated summary of these results is shown in Tab. II. It can be seen that, as expected, for all the $\tau_{3}$ examined the standard deviations of the estimates decrease continually as N increases. The estimates asymptotically approach the true values as N increases and the statistics are seen to improve. The occurrences of some exceptionally large standard deviations reflect the instability of the ratio estimate given by Equation 9 when the denominator is erroneously small with respect to the numerator due to data noise. Fig. 6. shows a simulated histogram for $N=1.5\times 10^{3}$ and $\tau_{3}=1.5\;\text{ns}$ when one such case occurs. It can be seen that the Δt>4 region of the histogram where o-Ps is assumed to dominate is sparsely populated, leading to large and inconsistent errors in the numerator and denominator of Equation 9. Controlling this variability will be commented on in Discussion Drawbacks and future work.

### Lifetime estimate versus cutoff and background

**Fig.7.**
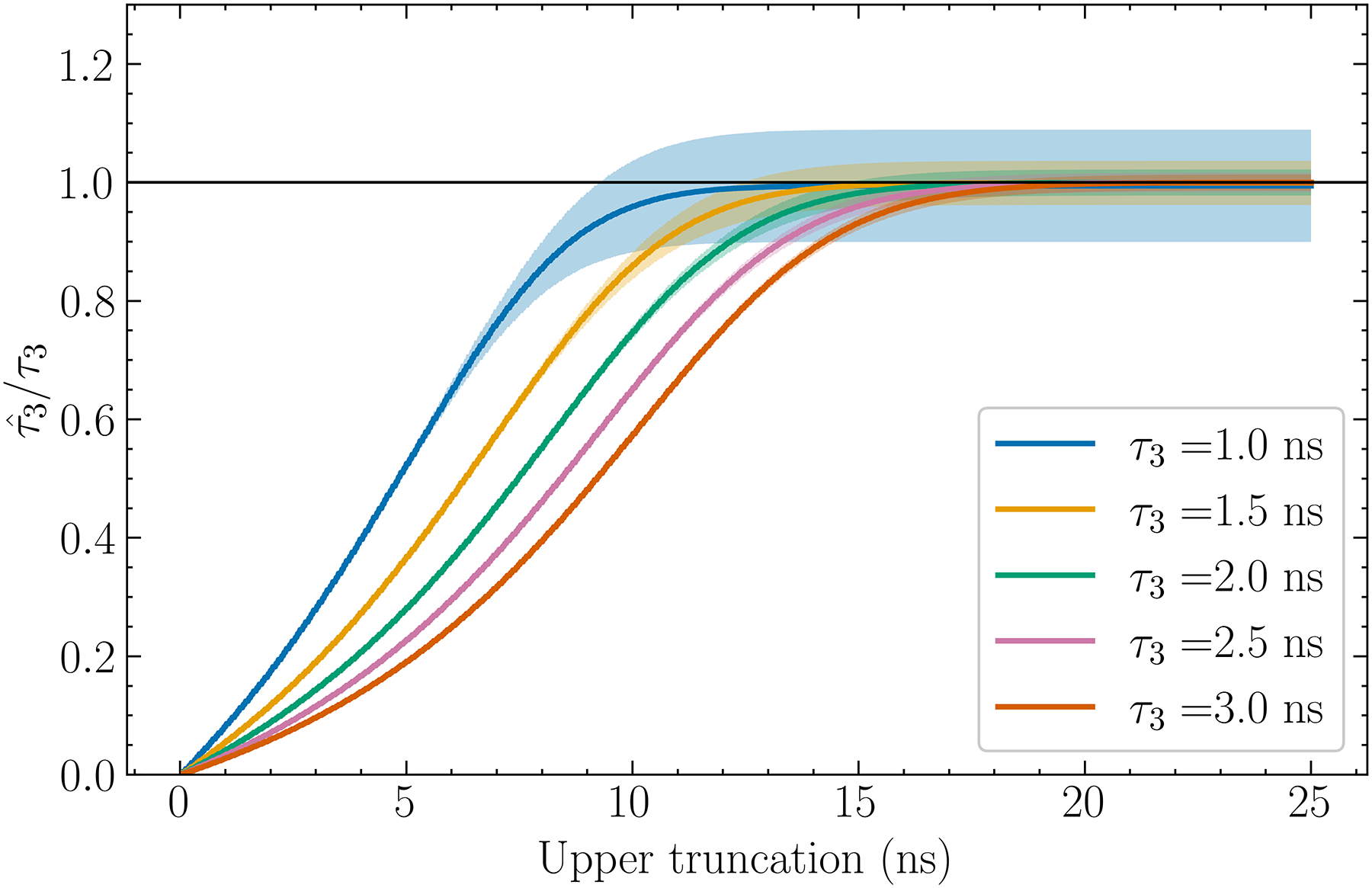
Estimated o-Ps lifetime with n = 11, N = 2 × 10^5^ as a function of the upper truncation threshold $\Delta {\text{t}}_{\text{up}}$. Note that here ${\overset{ˆ}{\tau }}_{3}/\tau_{3}$ is plotted. The horizontal line indicates the perfect estimate given by ${\overset{ˆ}{\tau }}_{3}/\tau_{3}=1$.

**Fig. 8.**
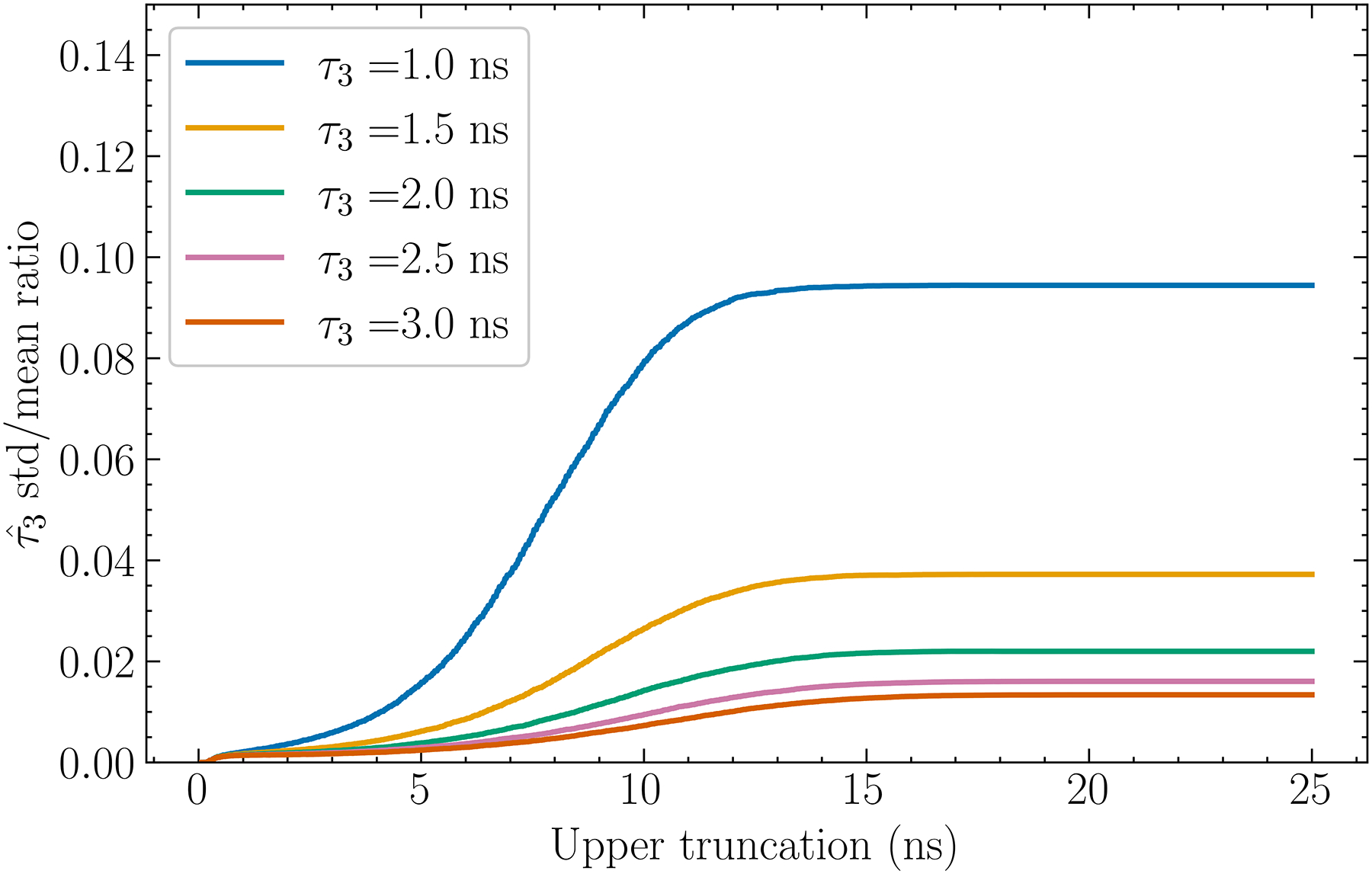
The ratio of the standard deviation to the mean of the curves from Fig. 7. The ratios are seen to decrease as the upper truncation decreases. The standard deviations about the mean for each plot point are calculated, however they are too small to be seen on this figure.

In an attempt to further alleviate the deleterious effects of noise, we also examined removing data above certain Δt. Fig. 7. shows that, for the case of n=11, as the upper truncation threshold $\Delta t_{\text{up}}$ is decreased, the standard deviation decreases, which is particularly evident for small $\tau_{3}$, whereas the bias increases in the form of underestimation. However, as shown in Fig. 8., the standard deviations relative to the distances between means increases as $\Delta t_{\text{up}}$ is lowered, at least for $\Delta t_{\text{up}}$ above ~10 ns. Therefore, at least for discrimination tasks, it is beneficial to apply truncation even though it introduces bias.

**Fig.9.**
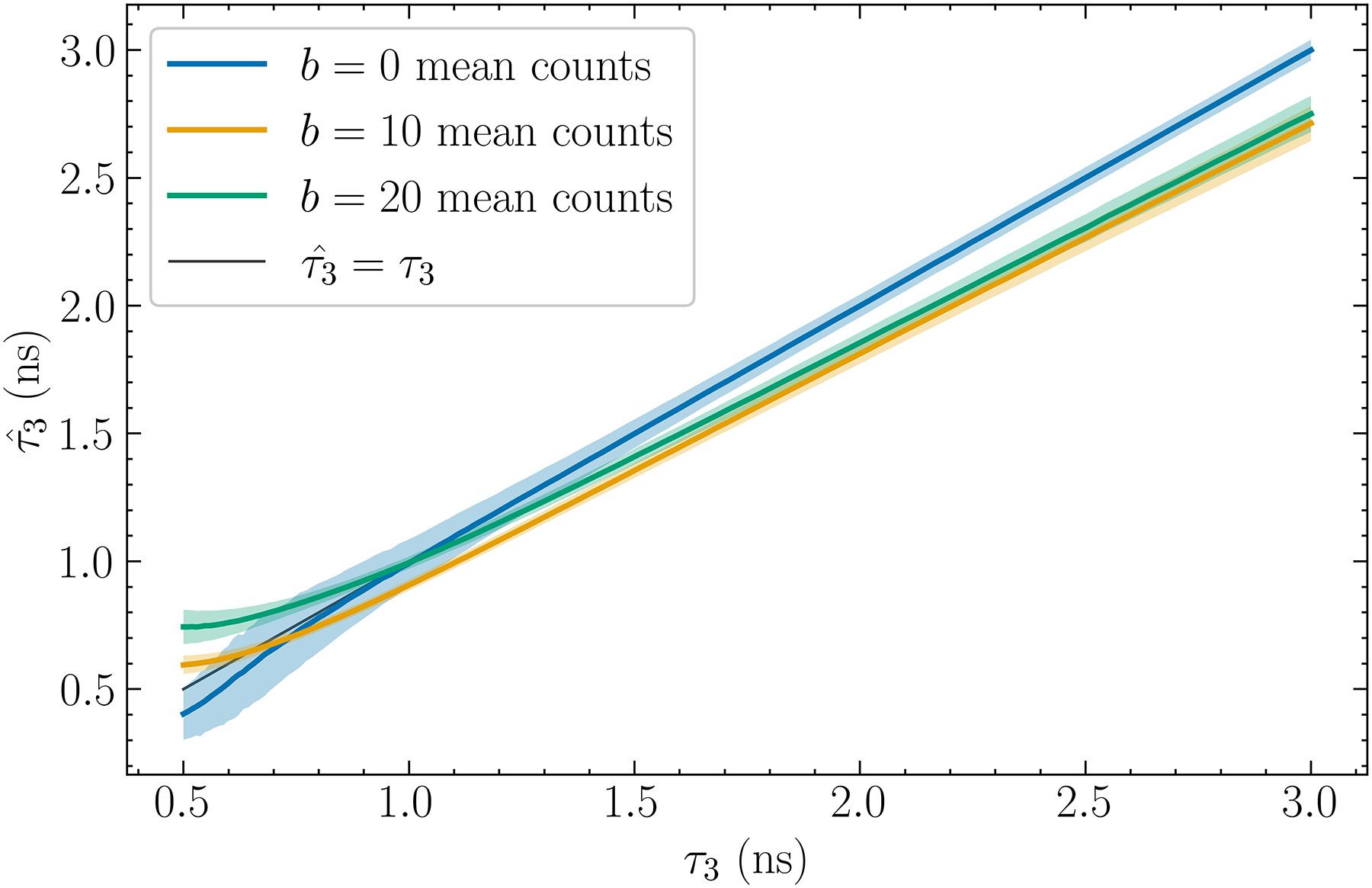
Estimated o-Ps lifetime versus the true value for three background levels b with $n=11,N=2\times 10^{5}$ and no upper truncation. The region close to $\tau_{3}=0.5\;\text{ns}$ may not be monotonic for some cases due to the curvature.

The presence of a nonzero background b increases data noise in two manners. First, for a given number of total events the number of events contributing to the signal is lowered. Second, although b may be estimated and subtracted from S(Δt), the noise associated with it remains in the data. Due to its Poisson nature, the variance of the associated noise is proportional to b. Fig. 9. shows the relationship between the estimate $\tau_{3}$ and the true $\tau_{3}$ for three background levels with $n=11,\;\text{N}=2\times 10^{5}$, and no upper truncation. The background is modeled as Poisson noise with means of b=0,10, and 20 counts.

### Monotonicity

We have observed that for discrimination tasks it is beneficial to allow for some bias if the std/mean ratio of the estimate decreases. For quantitative tasks, as long as ${\overset{ˆ}{\tau }}_{3}$ is related to $\tau_{3}$ monotonically in the mean, this observation remains true because we can correct for the bias in ${\overset{ˆ}{\tau }}_{3}$ by using, for example, a predetermined calibration curve. Such monotonicity is observed in both Fig. 7. and Fig. 9. Since curves in Fig. 3. do not cross one another, this monotonicity is also true when varying n. For b=0,10, and 20 mean counts, ${\overset{ˆ}{\tau }}_{3}$ remains monotonic with $\tau_{3}$ in the mean and is approximately linear for $\tau_{3}\geq 1.5\;\text{ns}$. Below approximately 0.5 ns, monotonicity may not hold for n=11 but this is well below the range for currently known biological values of $\tau_{3}$.

## DISCUSSION

### Effect of the number of events

It was observed that our moment-based method is significantly affected by the presence of Poisson noise, which comes with reduced event numbers. However, in the mean this method is still monotonic with the true value when several approaches for noise reduction were examined. A clinical PET system would produce a single histogram for each voxel (even though this is not necessary for estimating $\tau_{3}$ using our method). A typical scan may consist of approximately 185 MBq (5 mCi) of injected activity and a 64 mm^3^ voxel size (4 mm side length). Assuming the human body to be mainly composed of water with a total mass of 80 kg, it would have apporoximately 8 × 10^4^ cm^3^ of internal volume. The activity in this volume would then be 2.3 × 10^3^ Bq/cm^3^ and we might expect 148 Bq/voxel if there is a uniform activity distribution.

Assuming a per-voxel sensitivity of 1%, 1.48 counts would be collected each second. To obtain 10^3^ counts for each voxel, we would need to collect data for approximately 11.3 minutes. For reference, Moskal et al. reported human brain PLI data where the scan time was 10 minutes (after a standard radiotracer distribution waiting period), and the number of counts collected were 342 for the healthy brain tissue, 547 for the tumor, and 1119 for the salivary glands [3]. Additionally, above we have assumed a uniform radiotracer distribution. When using tumor-specific radiotracers, the radioactivity can be concentrated to tumor regions which will improve the statistics.

### Short o-Ps lifetimes *in vivo*

Thus far, the shortest published o-Ps lifetime value using a phantom was 1.8239 ns [4]. This was measured in fully O_2_ saturated water. In a more artificial environment, Stepanov et al. [11] bubbled oxygen gas through water and measured an o-Ps lifetime of 1.746 ns. With argon gas bubbled through water, however, a lifetime of 1.833 ns was measured. This is evidence that even extremely well-oxygenated *in vivo* environments will not have o-Ps lifetimes of less than 1.746 ns. In a recent review, Moskal and Stępień concluded that the mean biological o-Ps lifetime would be approximately 2 ns [1]. This moment-based method has been shown to produce viable results for lifetimes above approximately 1 ns, depending on noise, moment, and truncation. For lifetimes that are of the order of 1 ns, standard deviations for n > 15 (seen in Fig. 3.) grow rapidly, and estimations may no longer be monotonic.

### Drawbacks and future work

**Fig. 10.**
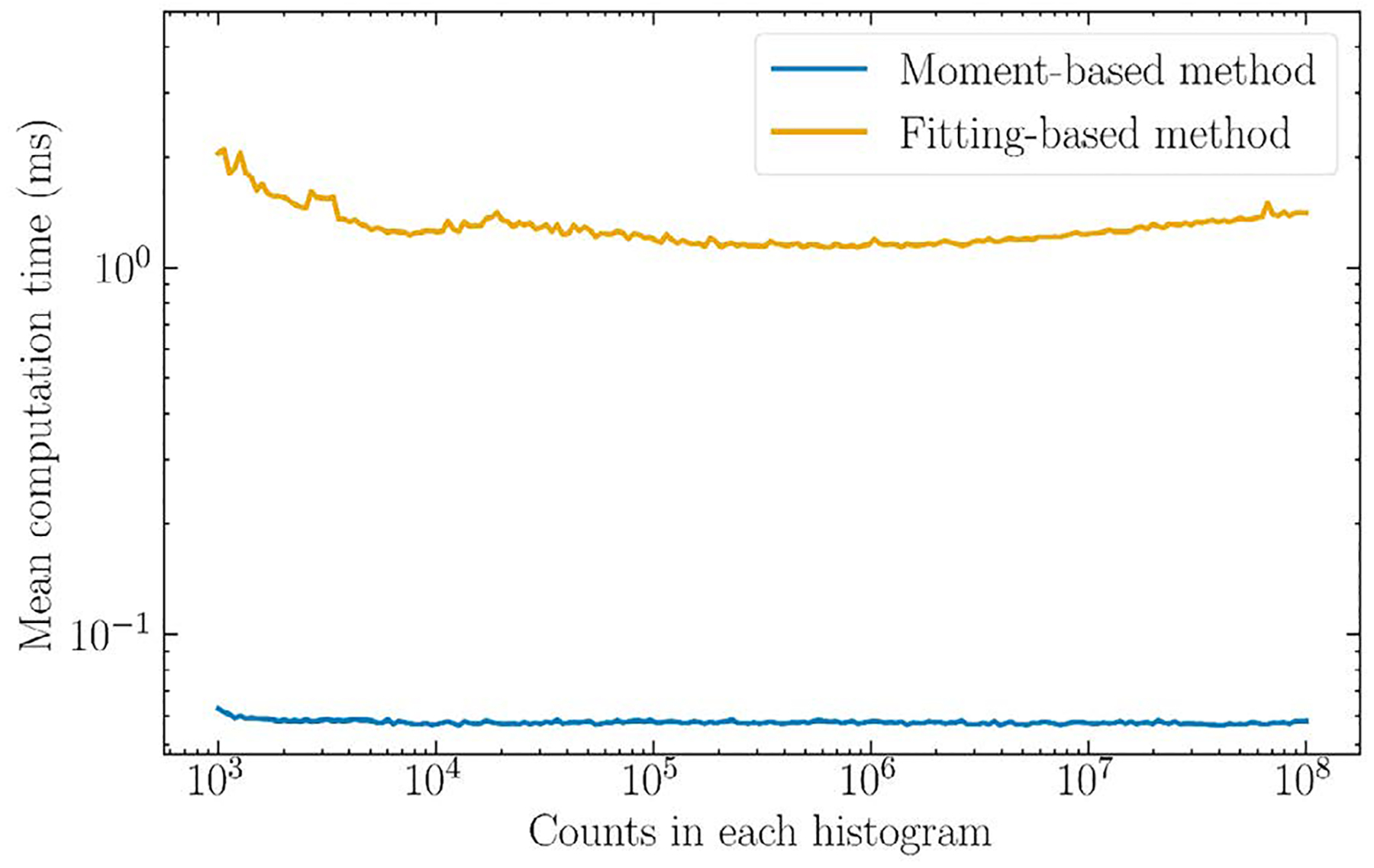
Mean computation time for 1 × 10^3^ simulations to estimate the o-Ps lifetime using the moment-based and single-exponential fitting-based methods. The chosen moment was n=10, and the histogram contained counts ranging from 1 × 10^3^ to 1 × 10^8^. The simulated o-Ps lifetime was 2 ns and the background was set to zero. For legibility the standard deviations are not shown.

A main drawback of this method is the response to noise. The standard deviations in Fig. 5. are a significant fraction of the estimate itself, even though the mean is stable. However, this method will be computationally less expensive to execute over an entire PET dataset than fitting-based methods, which may outweigh its precision for low-count cases. Fig. 10. shows a preliminary analysis of the computation time of the moment-based method compared to a single-exponential fitting-based method. The estimations were run times for each point, and the fitting-based method was computed using the scipy.optimize module in Python 3.11. The computer used to run the simulations had an Intel Core i7-7700 K central processing unit with 4 cores at 4.20 GHz, and 48 GB of random--access memory. Across all PALS spectrum histogram counts, the moment-based method mean computation time was 0.06 ms while the fitting-based method averaged 1.30 ms. The moment-based method was therefore found to be approximately 22 times faster in the mean. The standard deviations were large for the fitting--based method at 2 ms in the mean due to significant variations in a small number of estimations, while the standard deviations for the moment-based method were 0.02 ms in the mean.

Shibuya et al. previously calculated that approximately 3 × 10^8^ counts would be needed to estimate oxygen concentration to a precision of 10 mmHg [4]. This would correspond to three times as many counts as the extent of Fig. 5. At this point, the method has essentially reached its asymptote, minimum bias, and minimum standard deviation.

Additionally, this algorithm does not estimate the intensities of the positronium lifetime components, whereas a fitting-based method might. It is assumed that the o-Ps lifetime (i=3 term) dominates the sum in Equation 7, which allows the final estimation to be independent of the o-Ps component intensity. This algorithm was developed for *in vivo* o-Ps lifetime measurements specifically, where such assumptions may be made, and where the positronium intensities are of less concern.

**Fig. 11.**
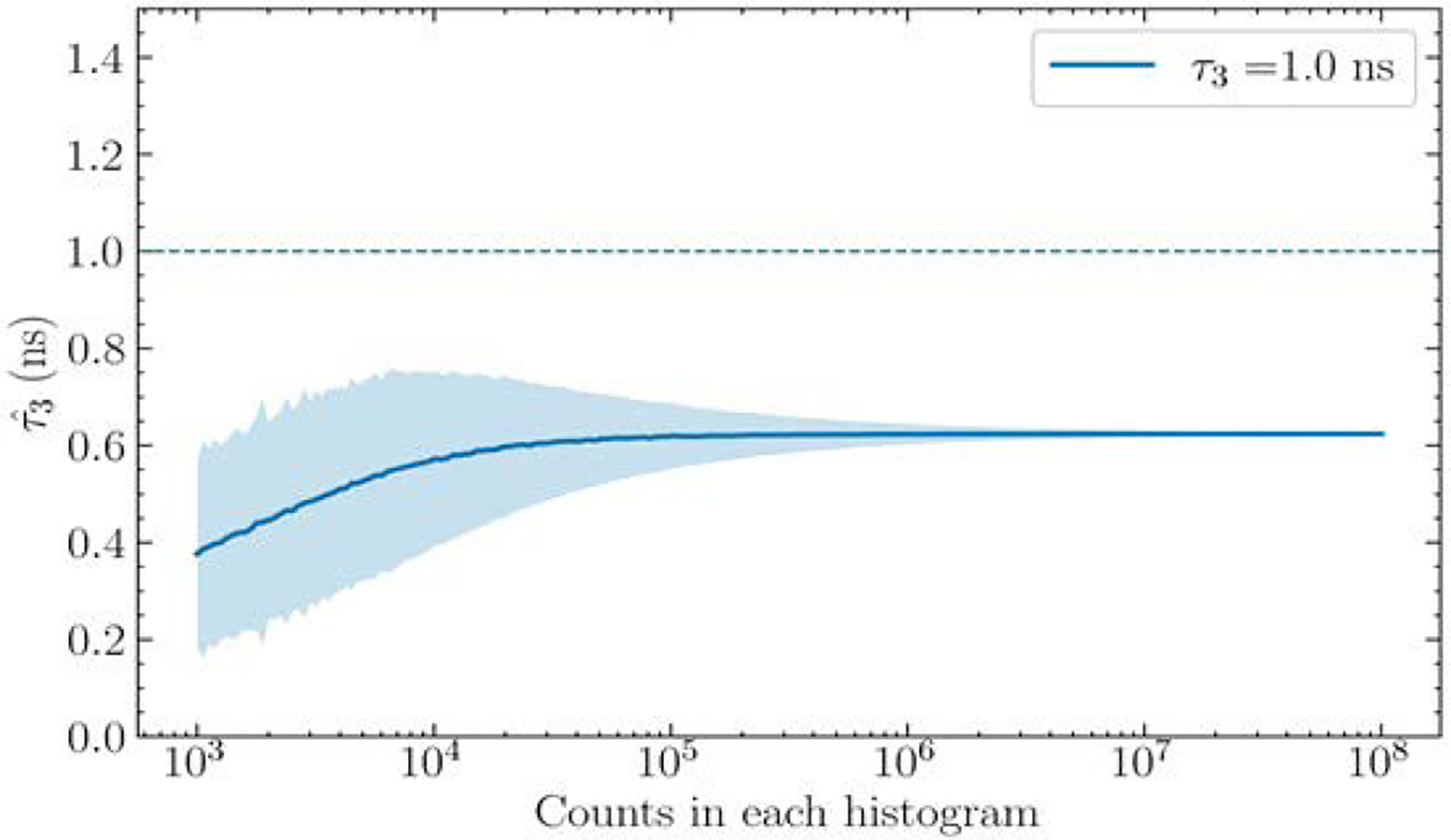
The estimate of the $\tau_{3}=1\;\text{ns}$ case from Fig. 5., however, with δ=0.3 to demonstrate how the standard deviation variability can be handled for small $\tau_{3}$ values.

To control variations in the standard deviation at low counts, a small positive number δ may be introduced, as shown in Equation 9. This results in an increased bias and decreased standard deviation, as seen in Fig. 11. Despite the increased overall bias in the asymptotic region (37.6% error), the curve remains monotonic and asymptotic, allowing for the bias to be easily corrected. For comparison, with 1 × 10^3^ histogram counts, the standard deviation decreased from 0.62 ns for δ=0 to 0.19 ns for δ=0.3, while the percentage error increased from 42.9% to 62.4% for the two cases, respectively. Future work on this estimation method will include its testing on full three-dimensional reconstruction, additional testing on its computational speed with respect to current estimation techniques, and further optimization of fitting parameters, such as the lower truncation point, the $e^{-st}$ term, smoothing of the histogram, and noise handling modifications. Finally, artificial intelligence-based methods for image denoising will be explored. A wide variety of approaches have been proposed [12, 13] and convolutional neural networks have been shown to excel at denoising particularly noisy PET images, such as those that may be encountered in PLI [14].

## CONCLUSIONS

In this report we present an analytical method to estimate the orthopositronium lifetime in PALS measurements. This method uses moments of the histogram of arrival time differences and employs an exponential weighting to mitigate numerical instability in the calculation of moments from noisy data. The moment-based method was characterized in this work, and it was shown to be a stable, monotonic estimate in most cases. For cases in which the standard deviation was large, modifications to the method may be employed. This method will continue development to control noise for cases with small statistical power and will be implemented and tested for three-dimensional PET images.

## Figures and Tables

**Tab. I.** Bias-minimizing estimates of Fig. 3, shown with the moment which minimized the bias. We again note that this moment-based method requires to be sufficiently large.

$\tau_{3}(\text{ns})$	ORDER n	${\overset{ˆ}{\tau }}_{3}$			
		MEAN (ns)	STD (ns)	STD/MEAN	% ERROR
1.0	11	0.99	0.094	0.095	0.654
1.5	11	1.50	0.056	0.037	0.096
2.0	12	2.00	0.051	0.025	0.008
2.5	10	2.50	0.041	0.016	0.001
3.0	10	3.00	0.037	0.012	0.009

**Tab. II.** Selected estimates from Fig. 5.

$\tau_{3}(\text{ns})$	${\overset{ˆ}{\tau }}_{3}(\text{ns})$			
	$N=10^{3}$	$N=10^{4}$	$N=10^{5}$	$N=10^{6}$
1.0	0.6 ± 0.6	0.9 ± 0.4	1.0 ± 0.1	0.997 ± 0.004
1.5	2 ± 16	1.49 ± 0.3	1.49 ± 0.08	1.498 ± 0.002
2.0	2.0 ± 0.8	2.0 ± 0.2	2.00 ± 0.06	2.000 ± 0.002
2.5	2.5 ± 0.6	2.5 ± 0.2	2.50 ± 0.06	2.500 ± 0.001
3.0	3.0 ± 0.6	3.0 ± 0.1	3.00 ± 0.06	3.000 ± 0.001

## Footnotes

1 In theory, p-Ps self-annihilation is permitted to produce n=2,4,6,⋯ coincidence photons but n=2 predominates. Likewise, o-Ps self--annihilation is permitted to produce = 3,5,7,⋯ coincidence photons but n=3 predominates. In direct annihilations, n≥2 photons can be created since any number of photons greater than n=2 can allow for momentum conservation.

2 Given the CRT in FWHM, if time measurements made by all channels are independent, the standard deviation of the single-channel time measurement of a TOF PET system is $\sigma_{1}=\sigma_{\text{TOF}}/\sqrt{2}$, where $\sigma_{\text{TOF}}=\text{CRT}/2.35$. The arrival-time difference is calculated by $\Delta t=\left(t_{511,1}+t_{511,2}\right)/2-t_{\gamma }$, where the $t_{511,\text{i}}$ components are the measured arrival times of the annihilation photons, and $t_{\gamma }$ is the measured arrival time of the prompt gamma. The standard deviation in Δt is therefore equal to $\sqrt{3}/2\sigma_{1}=\left(\sqrt{3}/2\right)\sigma_{\text{TOF}}$.

## APPENDIX

From $f_{n}(\lambda )=\mu_{n}\{\text{EXP}(t;\lambda )\}=\lambda \int_{0}^{\infty }\text{dt}\;{\text{t}}^{n}{\text{e}}^{-\lambda t}$, one can immediately derive

(10)
$$\frac{df_{n}(\lambda )}{d\lambda }=\frac{1}{\lambda }f_{n}(\lambda )-f_{n+1}(\lambda ).$$

Using Equation 5 in the above equation then yields

(11)
$$f_{n+1}(\lambda )=\frac{1}{\lambda }\frac{n!}{\lambda^{n}}+n\frac{(n+1)!}{\lambda^{n+1}}.$$

When n=0, Equation 5 also correctly yields $f_{0}(\lambda )=1$. Therefore, by induction Equation 5 is valid for n≥0. The function g(t;λ,σ)=EXP(t;λ)*N(t;σ) is known as the Exponential Modified Gaussian (EMG) and can be shown to equal

(12)
$$g(t;\lambda ,\sigma )=\lambda e^{-\lambda t}\;e^{\sigma^{2}\lambda^{2}/2}h(t;\lambda ,\sigma ),$$

where

(13)
$$h(t;\lambda ,\sigma )=\frac{1}{2}\left(1+\text{erf}\left(\frac{t-\sigma^{2}\lambda }{\sqrt{2}\sigma }\right)\right)$$

and erf(t) is the error function [15]. At large t,h(t;λ,σ)≈1 because erf(t)≈1. Therefore,

(14)
$$e^{-st}g(t;\lambda ,\sigma )\approx \frac{\lambda }{s+\lambda }e^{\sigma^{2}\lambda^{2}/2}\;\text{EXP}(t;s+\lambda ).$$

Note that, in evaluating $\mu_{n}\left\{f\left(t\right)\right\}=\int \;dt{\;t}^{n}f(t)$, the term $t^{n}$ in the integral progressively diminishes the contribution of f(t) at small t as n increases. Therefore, for sufficiently large n, one can use the approximation given by Equation 14 to evaluate $\mu_{n}\left\{e^{-st}g(t;\lambda ,\sigma )\right\}$, which then yields Equation 6.

## LIST OF ABBREVIATIONS

CRT: coincidence resolving time
DA: direct annihilations
FWHM: full width at half maximum
o-Ps: orthopositronium
PALS: positron annihilation lifetime spectroscopy
PET: positron emission tomography
PLI: positronium lifetime imaging
p-Ps: parapositronium
Ps: positronium
std: standard deviations
TOF: time-of-flight
